# The impact of antenatal massage practice on intrapartum massage application and their associations with the use of analgesics during labour: Sub-analysis of a randomised control trial

**DOI:** 10.1186/s12884-022-04743-7

**Published:** 2022-05-18

**Authors:** Chit Ying Lai, Margaret Kit Wah Wong, Wing Hung Tong, Kam Yan Lau, Suk Yin Chu, Agnes Mei Lee Tam, Lai Ling Hui, Terence T. H. Lao, Tak Yeung Leung

**Affiliations:** 1 Department of Obstetrics and Gynaecology, The Chinese University of Hong Kong, Shatin, Hong Kong; 2 Department of Obstetrics and Gynaecology, Prince of Wales Hospital, Shatin, Hong Kong; 3 Department of Obstetrics and Gynaecology, Kwong Wah Hospital, Kowloon, Hong Kong; 4 Department of Paediatrics, The Chinese University of Hong Kong, Shatin, Hong Kong

**Keywords:** Antenatal massage compliance, Intrapartum massage application, Pethidine, Epidural analgesia

## Abstract

**Background:**

Massage during labour is one form of intrapartum non-pharmacological pain relief but it is not known whether the frequency of practicing these massage techniques among couples during the antenatal period could enhance the effectiveness of intrapartum massage. This study was to evaluate the association between compliance of antenatal massage practice with intrapartum application and their impact on the use of analgesics during labour.

**Methods:**

This was a sub-analysis of a childbirth massage programme which was carried out in two public hospitals with total births of around 8000 per year. Data from women who were randomized to the massage group were further analysed. After attending the pre-birth training class on massage at 36 weeks gestation, couples would be encouraged to practice at home. Their compliance with massage at home was classified as good if they had practiced for at least 15 minutes for three or more days in a week, or as poor if the three-day threshold had not been reached. Application of intrapartum massage was quantified by the duration of practice divided by the total duration of the first stage of labour. Women’s application of intrapartum massage were then divided into above and below median levels according to percentage of practice. Logistic regression was used to assess the use of epidural analgesia or pethidine, adjusted for duration of labour and gestational age when attending the massage class.

**Results:**

Among the 212 women included, 103 women (48.6%) achieved good home massage compliance. No significant difference in the maternal characteristics or birth outcomes was observed between the good and poor compliance groups. The intrapartum massage application (median 21.1%) was inversely associated with duration of first stage of labour and positively associated with better home massage practice compliance (*p* = 0.04). Lower use of pethidine or epidural analgesia (OR 0.33 95% CI 0.12, 0.90) was associated with above median intrapartum massage application but not antenatal massage compliance, adjusted for duration of first stage of labour.

**Conclusions:**

More frequent practice of massage techniques among couples during antenatal period could enhance the intrapartum massage application, which may reduce the use of pethidine and epidural analgesia.

**Trial registration:**

(CCRBCTR) Unique Trial Number CUHK_ CCRB00525.

## Background

Women rank labour pain as the topmost painful life experience [1]. Fear and anxiety can induce muscle tension and further intensify the perception of pain [2]. Pharmacological pain relief, the most frequently used form of labour pain management, is effective but costly and not free of side effects [3, 4]. The most effective pharmacological pain relief is epidural analgesia, the use of which may cause numbness and limit the patients’ mobilization [5]. Thus women are ambivalent towards the use of epidural analgesia despite its effectiveness in pain control, as they are often disappointed with themselves for not being able to accomplish a natural birth [6]. Hence, non-pharmacological analgesic methods become the only alternative to women who want to maintain their well-being throughout the labour process [7].

One of the most commonly studied non-pharmacological pain relief methods is massage. Massage works through the application of pressure on parts of the body to block the transmission of pain impulses to the brain, and may improve blood flow and oxygenation of tissues [8]. However, its reported analgesic effects during labour are inconsistent. A number of randomized controlled trials have demonstrated the efficacy of massage in reducing or delaying the use of epidural analgesia [9, 10], lowering pain intensity [11–15] and reducing the incidence of depressed mood [16]. Another study reported no difference in both pain score and the use of intrapartum analgesia with the use of massage [17]. However, all these studies have focused on the impact of intrapartum massage on their respective outcome measures. These studies either did not include antenatal massage practice as their intervention [11–15] or have only assessed the effect of pre-birth massage training on labour outcomes [9, 16, 17].

There are only three reported trials on pre-birth training of massage techniques, but none of them had looked into the relationship between antenatal massage practice with the effectiveness of intrapartum pain relief or the need to use other forms of analgesia [9, 16, 17]. We have previously conducted a clinical trial in which healthy, pregnant, nulliparous women were randomized to a pre-birth massage training group and a control group (who were not trained in massage techniques and under usual care only), and found lower intrapartum use of pharmacological pain relief modalities in the pre-birth massage training group [18]. A sub-analysis was conducted aiming firstly to examine, within the pre-birth training group, the association between compliance of antenatal massage practice with intrapartum massage application; and secondly their impact on the use of pain analgesics during labour.

## Methods

This is a sub-analysis of our Randomized Controlled Trial (RCT) conducted on 233 healthy low risk nulliparous women of Chinese ethnicity who carried a singleton pregnancy and were planned for vaginal delivery. Those women who indicated that no partner would be present during birth, and those who actually delivered in hospitals other than the study hospitals were excluded from the study. The details of the massage programme has been described before [18]. In brief, the women were randomized into the massage or the control group (with standard care) at 36 weeks of gestation. Women allocated to the massage group received a 2-hour pre-birth massage training session with their partners from 36 weeks gestation. The programme content was based on the childbirth massage programme developed by Linder Kimber at the Childbirth Essentials, which consists of multiple components including massage, breathing and visualization [19]. The trainers were midwives accredited as trainers of the LK Massage Programme. Couples who consented to the study were taught relaxation and pain relief massage techniques together with controlled breathing and visualization. Relaxation techniques included massage over the temple area, arms, upper legs, whole back, and shoulders. Pain relief techniques included massage over the sacral region as well as practicing controlled breathing and visualization during the massage. Participants were advised to adopt various positions, such as lying in bed or maintaining an upright or sitting position, according to different types of massage received. They were advised to practice for at least 15 minutes daily; three times a week, and preferably before bedtime. The participants were instructed to record the time spent on practice each day until the day of delivery and to submit this practice record to research staff upon admission for birth.

### Exposure – Antenatal massage compliance and intrapartum massage application

Antenatal massage practice was quantified by the number of days with practice for at least 15 minutes/day, between the massage class and delivery. The women’s antenatal massage compliance was classified as ‘good’ if they had on average practiced the massage techniques for three or more days in a week. This demarcation was made with reference to Kimber’s study in 2008, which concluded that 46.7% women could practice three or more times per week [17]. Breathing and visualization were difficult to quantify and therefore not recorded as a compliance criteria in this study. Nevertheless, couples were encouraged to practice all these programme components when performing massage at home.

Intrapartum massage application was measured by the duration (in minutes) of massage during the first stage of labour and was recorded by midwives. Application of intrapartum massage was calculated as proportion of first stage of labour with massage, i.e. the duration of massage (in minutes) divided by the total duration of the first stage of labour (in minutes). The level of intrapartum massage application is then categorised into above, at or below median respectively. Again, the components of controlled breathing and visualisation were not assessed and recorded but couples would be reminded to practice during the intrapartum massage application.

### Outcome

Use of epidural analgesia or pethidine injection during labour.

### Statistical analysis

Maternal baseline characteristics by massage practice at home and during labour were compared using chi-square tests (for categorical variables), t-test (for continuous variables with normal distribution) and Mann-Whitney U Test (for continuous variables with skewed distribution).

Multiple logistic regression was used to assess the associations of antenatal massage practice and intrapartum massage compliance with the use of pethidine or epidural analgesia. The statistical analyses were performed using a commercial statistical package SPSS version 26 and the R version 4.0.1 (R Foundation for Statistical Computing, Vienna, Austria).

## Results

### Antenatal massage compliance

**Fig. 1:**
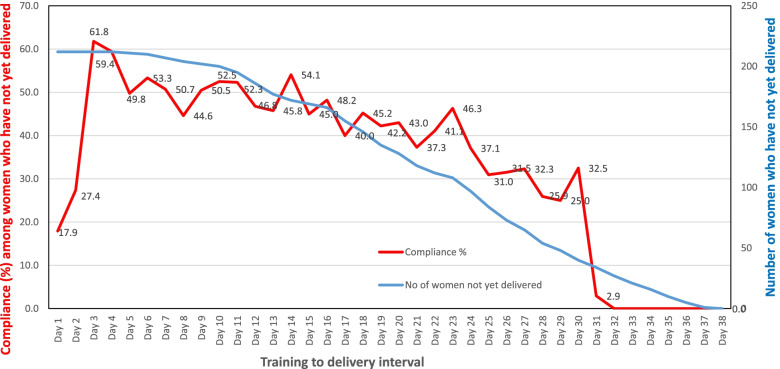
Percentage of women who practiced antenatal massage at home for at least 15 minutes per day, starting from completion of massage training till childbirth

From the total 233 women randomized to the massage group in the RCT, 21 did not return their home practice record, leaving 212 in the final analysis. The participants attended massage class from 36.0 to 38.1 gestational weeks (median 36.4 weeks), and delivered at 36.9 to 41.6 weeks gestation (median 39.6 weeks). The duration from massage class to delivery ranged from 5 days to 38 days. Figure 1 shows the numbers of women who have not yet delivered and the proportion of women that practiced massage for at least 15 min a day, which reached a maximum of 61.8% on day 3. This percentage then declined gradually to about 45% after 2 weeks, 30% on day 30, and dropped steeply afterwards. The massage practice of 103 out of 212 women (48.6%) were classified as good compliance based on our pre-set criteria. Table 1 shows that the maternal characteristics between women with good and poor compliance were similar in terms of distributions of maternal age, BMI, education levels, occupation and family income. The women who practiced good compliance attended the massage class at slightly later gestation (median 36.6 weeks vs 36.4 weeks; *p* = 0.01), but there was no significant difference in terms of the median gestation at delivery or the need of induction of labour (Table 1). The median duration from massage class to delivery was 21 days, which was the same in women who practiced good compliance and those who did not (Table 1).

**Table 1:** Comparison of maternal characteristics between women with good compliance and those with poor compliance of antenatal massage practice

	Antenatal massage practice		
	^**a**^Good compliance ***N*** = 103	Poor compliance ***N*** = 109	***p***-values
**Maternal Characteristics**			
Age mean (**+/−**SD)	31.2 (3.8)	31.3 (3.6)	0.98
Height mean (+/−SD, cm)	157.9 (5.2)	158.9 (5.7)	0.21
BMI at first booking mean (+/−SD)	26.4 (3.0)	26.4 (3.3)	0.99
Education (%)			0.79
• Secondary	30 (29.1)	30 (27.5)	
• Tertiary or above	73 (70.9)	79 (72.5)	
Occupation (%)			0.31
• Housewives	20 (19.4)	20 (18.3)	
• Nonprofessional	60 (58.3)	73 (67.0)	
• Professional	23 (22.3)	16 (14.7)	
Income (%)			0.29
• < HK$20000	12 (11.7)	29 (18.3)	
• HK$20000 to HK$40000	51 (49.5)	55 (50.5)	
• > HK$40000	40 (38.8)	34 (31.2)	
Gestation at massage class median (IQR)	36.6 (36.0, 38.1)	36.4 (36.0, 37.7)	0.01
**Pre-labour Characteristics**			
Gestation at delivery median (IQR)	39.7 (36.9,41.4)	39.6 (37.1,41.6)	0.43
Median (IQR) interval between massage class to delivery (days)	21 (15, 26)	21 (14.5, 28)	0.56
Induction of labour (%)	51 (49.5)	49 (44.5)	0.51
Reasons for induction of labour			0.44
• Post date	8 (15.7)	13 (26.5)	
• Ruptured membranes	20 (39.2)	13 (26.5)	
• Placental degeneration	9 (17.6)	6 (12.2)	
• Infection risk	7 (13.7)	10 (20.4)	
• Fetal problem	7 (13.7)	7 (14.4)	

^**a**^Good compliance was defined as antenatal massage >15mins/day for 3 days or more per week

### Intrapartum massage application

Intrapartum massage application was calculated by dividing the duration of massage by the duration of the first stage of labour. The median duration of the first stage of labour was 405 minutes (IQR 260, 624), and the median duration of intrapartum massage was 100 minutes (IQR 35, 195). The longer the first stage of labour, the longer the total duration couples performed intrapartum massage (Spearman’s coefficient rho = 0.39, *p* < 0.0001). The overall median application % of intrapartum massage was 21.1% (IQR 10.3, 46.6), and hence 110 women and 102 women were classified as above and below median application respectively. Among the 90 women with first stage of labour lasting 6 hours or less, their median massage application was 28.1% (IQR 9.4, 64.9), which was higher than that of the 89 women (21.5%; IQR 13.9, 39.3) and 33 women (20.1%; IQR 8.1, 32.3) whose first stages were respectively > 6–12 hours and > 12 hours, although these differences were not statistically significant. No significant difference in the maternal characteristics and birth outcomes was found with respect to the degree of intrapartum massage application (Table 2).

**Table 2:** Comparison of maternal characteristics and birth outcomes between intrapartum massage application

	^**a**^Intrapartum massage application (Median: 21.1% IQR 10.3–46.6)		
	> Median ***N*** = 110	=/< Median ***N*** = 102	***P*** values
**Maternal Characteristics**			
Age mean (**+/−**SD)	31.3 (3.8)	31.2 (3.7)	0.98
Height mean (+/−SD, cm)	158.6 (5.2)	158.2 (5.8)	0.60
BMI at first booking mean (+/−SD)	26.3 (3.2)	26.5 (3.1)	0.65
Education (%)			0.73
• Secondary	30 (27.3)	30 (29.4)	
• Tertiary or above	80 (72.7)	72 (70.6)	
Occupation (%)			0.20
• Housewives	18 (16.4)	22 (21.6)	
• Nonprofessional	67 (60.9)	66 (64.7)	
• Professional	25 (22.7)	14 (13.7)	
Income (%)			0.55
• < HK$20000	14 (12.7)	18 (17.6)	
• HK$20000 to HK$40000	58 (52.7)	48 (47.1)	
• > HK$40000	38 (34.5)	36 (35.3)	
**Birth Outcomes**			
Augmentation of labour	15 (14.6)	10 (9.2)	0.22
Reasons for augmentation of labour			0.42
• Poor progress	5 (33.3)	6 (60.0)	
• Inadequate uterine contractions	5 (33.3)	2 (20.0)	
• Infection risk	5 (33.3)	2 (20.0)	
Duration of first labour			0.58
• < = 6 hours	50 (45.5)	40 (39.2)	
• > 6–12 hours	45 (40.9)	44 (43.1)	
• > 12 hours	15 (13.6)	18 (17.6)	
Mode of delivery			0.62
• Normal vaginal delivery	87 (79.1)	75 (73.5)	
• Instrumental delivery	12 (10.9)	15 (14.7)	
• Caesarean section	11 (10.0)	12 (11.8)	

^**a**^Intrapartum application was defined as the % of first stage of labour with massage practice

Table 3 shows the association of antenatal massage practice with intrapartum massage application during first stage of labour. Women with good intrapartum massage application were statistically significantly associated with good antenatal massage compliance overall (*p* = 0.04).

**Table 3:** Association of antenatal massage practice with intrapartum massage application during first stage of labour

	Intrapartum massage application (Median: 21.1% IQR 10.3–46.6)		
Antenatal massage practice	Above median ***N*** = 110 (%)	At or below median ***N*** = 102 (%)	***p*** value
**Overall compliance**			0.04
^a^Good (*N* = 103)	61 (55.5)	42 (41.2)	
Poor (*N* = 109)	49 (44.5)	60 (58.8)	

^a^Good compliance was defined as antenatal massage >15mins/day for 3 days or more per week

### Associations of antenatal massage practice and intrapartum massage application with the use of pethidine or epidural analgesia

Twenty two (10.4%) women requested and used either pethidine or epidural analgesia for intrapartum pain relief (Table 4). The proportion of women using these two forms of pharmacological analgesia was not significantly different between the groups with good versus poor antenatal massage compliance, but was related to the duration of the first stage of labour and the application of intrapartum massage (Table 4). Only 4 out of 90 women whose labour duration lasting 6 hours or less used pethidine or epidural analgesia, which was significantly lower than that among those with labour duration longer than 12 hours (OR 0.10 CI 0.03, 0.38). Furthermore, the proportion of women who gave birth within a normal labour duration (within 6 to 12 hours) received fewer doses of pethidine or epidural analgesia than those whose duration of labour was more than 12 hours (OR 0.25 CI 0.09, 0.71). Above median intrapartum massage application was associated with a 68% lower use of pethidine or epidural analgesia (OR 0.33 95% CI 0.12, 0.89), adjusted for potential confounders including the duration of labour, and gestational age when attending massage class (Table 4).

**Table 4:** Use of pharmacological pain relief methods by antenatal massage compliance, intrapartum massage application, and duration of labour

		Use of Pethidine/Epidural		
	n	*N* = 22 (%)	OR (95% CI)	*p*-value for OR
^**a**^**Antenatal massage compliance**				
• Good (>15mins for 3 days or more per week)	103	11 (50)	0.73 (0.28, 1.91)	0.52
• Poor (otherwise)	109	11 (50)	1.00	
^**a**^**Intrapartum massage application (% labour duration with massage)** (median 21.1% IQR 10.3–46.6)				
• Above median	110	6 (27.3)	0.33 (0.12, 0.90)	0.03
• Below median	102	16 (72.7)	1.00	
^**a**^**Duration of first stage labour (hours)**				
• = < 6	90	4 (18.2)	0.10 (0.03, 0.38)	< 0.001
• > 6 to 12	89	8 (36.4)	0.25 (0.09, 0.71)	0.01
• > 12 (referent group)	33	10 (45.5)	1.00	

^**a**^Adjusted for duration of labour (below 6 hours, 6 to 12 hours or above 12 hours) and gestational age when attending massage class

## Discussion

This is the first study to look into the impact of antenatal massage practice on intrapartum massage application as well as their association with the use of pharmacological analgesia. The results showed that compliance to antenatal massage practice was positively correlated with intrapartum massage application, which in turn was associated with a lower use of pethidine or epidural analgesia. However, antenatal massage compliance alone was not associated with the use of these two analgesics during labour. It was observed that women who practiced antenatal massage for 15 minutes, 3 days a week, from 36 weeks gestation onwards, applied massage more during labour. About half of the women who participated in this study reached such compliance of antenatal practice, which was similar to a previous smaller-scale study reporting a similar compliance of 10 minutes of practice, 3 days a week [17]. Around 50% of women gave birth by day 21 after the massage class and their compliance maintained at 40% or above within these 21 days. The study suggested that starting the massage practice a few weeks before birth may allow couples to become more familiar with applying the massage skills. Further studies are required to determine the optimal time to commence antenatal massage practice in order to achieve optimal application during the intrapartum period.

Greater intrapartum massage application was associated with lower use of pethidine or epidural analgesia during labour. However, the compliance to antenatal massage alone was not associated with a lower use of pethidine or epidural analgesia during labour, the use of which is more likely due to stronger factors such as the duration of labour. Women experiencing shorter labour duration in our study had particularly lower consumption of pethidine and epidural analgesia. This may be due to the fact that women experiencing a shorter labour duration can manage without analgesics since massage was adequate. A longer labour duration may decrease the participants’ pain threshold and partners might became fatigued with massage application, thereby increasing the need of pethidine or epidural analgesia. This is also consistent with the primary analysis of our RCT, which showed an increased use of pharmacological pain relief modalities in the control group when compared to the massage group [18]. Duration of labour is a predicting factor that affects the use of analgesia in labour in addition to parity and experience of previous labour [20]. The current study group is exclusively nulliparous and therefore duration of labour might be the only strong predicting factor in using pain analgesics.

This study shows that the use of pharmacological pain relief does not differ according to antenatal massage compliance, implying that an on-site massage class right before labour should still be considered and encouraged for couples who are keen to avoid the use pethidine or epidural analgesia. Nevertheless, since overall good antenatal massage compliance is related to subsequent greater application of intrapartum massage, a structured pre-birth childbirth massage programme should still be considered in equipping couples with the skills of massage and better preparation for labour.

### Limitations of the study

The median intrapartum massage application proportion was 21.1% which indicated half of the couples in the massage group spending around one fifth of the labour time applying massage. The optimal duration of massage during labour cannot be concluded. It is noted that some local practice may be barriers to the massage practice. For example, routine intrapartum continuous fetal heart monitoring might limit women’s mobility and interfere with some massage techniques applied in an upright posture. Revisiting the practice of continuous fetal heart monitoring is necessary in future. In healthy low risk women, intermittent auscultation should be considered or wireless cardiotocographic monitoring can be used when deemed necessary. Another potential barrier concerns the availability of partners who are not allowed to be present during emergency situations involving other parturients in the labour ward. However, the continuous presence of the partner throughout the labour process could be an important factor for the success of the intrapartum massage by giving both practical and emotional support to the women [21–23]. To support and enhance labour companionship is another important factor to maximize the application of massage during labour to a greater extent.

The current study is the largest study to look into both antenatal compliance and intrapartum application of the massage programme. It is noted that this current study focused on the compliance of massage only and did not evaluate other components of the massage programme such as controlled breathing and visualization. There are two main reasons. First, the massage was practiced in the presence of the women’s partners, while women could practice controlled breathing and visualisation on her own at any time. Hence it is more objective to assess the compliance to massage with a measurable duration. Second, our study aims at investigating the impact of the massage programme on the intrapartum massage application by the women’s partners. In this regard, the partners’ skill, experience and compliance, which were expected to be acquired during the antenatal period is the foremost influential factor when compared to maternal practice of breathing and visualisation. Hence, this study cannot determine whether maternal practice of controlled breathing and visualisation will have potential impact on the programme. A thorough evaluation of the childbirth massage programme should be considered in future and a system of breathing and visualization quantification should be established.

Intrapartum massage application was associated with lowered use of analgesics during labour. Whether it is equally effective by performing bedside practice alone during labour or home practice during antenatal period to enhance massage skills beforehand remains uncertain. Future randomized controlled trials on antenatal and intrapartum massage can be considered to explore the efficacy of the models of practice.

## Conclusion

Intrapartum massage practice, rather than antenatal massage, was associated with the reduced use of pethidine or epidural analgesia during labour. However, antenatal massage practice may enhance the intrapartum massage application, suggesting its indirect value in relieving labour pain. Non-pharmacological labour assistance program appears to be an option associated with the lesser use of the pethidine or epidural analgesia during labour.

## Data Availability

The datasets used and analyzed during current study are available from the corresponding author on reasonable request.

## Abbreviations

LK Massage: Linda Kimber Massage
RCT: Randomised controlled trial
